# Assessing Treatment Fidelity within an Epilepsy Randomized Controlled Trial: Seizure First Aid Training for People with Epilepsy Who Visit Emergency Departments

**DOI:** 10.1155/2019/5048794

**Published:** 2019-02-03

**Authors:** Adam J. Noble, Darlene Snape, Leone Ridsdale, Myfanwy Morgan, Sarah J. Nevitt, Steve Goodacre, Anthony Marson

**Affiliations:** ^1^Department of Health Services Research, University of Liverpool, Liverpool, UK; ^2^Department of Basic and Clinical Neuroscience, King's College London, London, UK; ^3^Institute of Pharmaceutical Science, King's College London, London, UK; ^4^Department of Biostatistics, University of Liverpool, Liverpool, UK; ^5^School of Health and Related Research, Sheffield University, Sheffield, UK; ^6^Department of Molecular and Clinical Pharmacology, University of Liverpool, Liverpool, UK

## Abstract

**Purpose:**

To measure fidelity with which a group seizure first aid training intervention was delivered within a pilot randomized controlled trial underway in the UK for adults with epilepsy who visit emergency departments (ED) and informal carers. Estimates of its effects, including on ED use, will be produced by the trial. Whilst hardly ever reported for trials of epilepsy interventions—only one publication on this topic exists—this study provides the information on treatment fidelity necessary to allow the trial's estimates to be accurately interpreted. This rare worked example of how fidelity can be assessed could also provide guidance sought by neurology trialists on how to assess fidelity.

**Methods:**

53 patients who had visited ED on ≥2 occasions in prior year were recruited for the trial; 26 were randomized to the intervention. 7 intervention courses were delivered for them by one facilitator. Using audio recordings, treatment “adherence” and “competence” were assessed. Adherence was assessed by a checklist of the items comprising the intervention. Using computer software, competence was measured by calculating facilitator speech during the intervention (didacticism). Interrater reliability was evaluated by two independent raters assessing each course using the measures and their ratings being compared.

**Results:**

The fidelity measures were found to be reliable. For the adherence instrument, raters agreed 96% of the time, PABAK-OS kappa 0.91. For didacticism, raters' scores had an intraclass coefficient of 0.96. In terms of treatment fidelity, not only were courses found to have been delivered with excellent adherence (88% of its items were fully delivered) but also as intended they were highly interactive, with the facilitator speaking for, on average, 55% of course time.

**Conclusions:**

The fidelity measures used were reliable and showed that the intervention was delivered as attended. Therefore, any estimates of intervention effect will not be influenced by poor implementation fidelity.

## 1. Introduction

International evidence shows people with epilepsy (PWE) frequently utilise emergency health services [1–3]. In the UK, up to 20% of PWE visit a hospital emergency department (ED) each year. In 2015/16, this cost the UK National Health Service (NHS) ~£70 million [4, 5]. Costs are high because ≤60% of PWE reattend ED within 12 months [6] and because half of the PWE visiting EDs are admitted to the hospital [7–9].

Emergency care for epilepsy can be appropriate and even life-saving. Most PWE attending EDs do not, though, attend for such reasons [10, 11]. Rather, most have known epilepsy and have experienced an uncomplicated seizure. Guidelines state that such seizures can be managed without medical attention by PWE and their family and friends [12, 13].

Reducing unnecessary emergency visits to the hospital by PWE is potentially important for service users since such visits can be inconvenient, do not typically lead to extra support [10], and there may be iatrogenic harms [14]. Reducing emergency visits has also been identified as one way health services can generate savings and manage demand [15]. To date, it has not been clear though how reductions can be achieved [16, 17].

One possibility is offering PWE and their carers an intervention to improve their confidence and ability to manage seizures. It has long been known that models of care within the UK and beyond fail to equip *all* PWE with the knowledge and skills needed to self-manage [18–22]. As a consequence, some PWE utilise ED for clinically unnecessary reasons [23–25].

As no such intervention was available [26, 27], we worked with PWE, carers, and health professionals to develop one [28]. The resulting intervention—titled “Managing Seizures: Epilepsy First Aid Training, Information and Support”—is a group-based psychoeducational intervention that lasts ~4 hours and which is delivered by a single facilitator.

It aims to improve recipients' understanding of when emergency attention is and is not required and how to manage postictal states and risk. Participants receive information, watch videos, and are asked to engage in a variety of activities that seek to elicit and challenge any inaccuracies or fears they have about seizures.

As the intervention consists of various interconnecting parts, it comprises a “complex intervention” [29]. The intervention and its rationale have previously been described in full [28, 30].

A multicentre pilot randomized controlled trial (RCT) (ISRCTN13 871 327) is currently comparing the intervention alongside treatment as usual to treatment as usual alone [28]. It will help determine the optimal design of a definitive trial. This includes providing estimates of the intervention's effect on the proposed primary outcome measure, which is the use of ED over the 12 months following randomization. Secondary outcomes include quality of life and knowledge of seizure first aid.

To permit accurate interpretation of such estimates, information on implementation fidelity—that is, the degree to which the intervention was delivered as intended within the trial and with what sort of consistency—is required [31]. Such information therefore helps avoid interpretation errors, such as falsely attributing the absence of a significant effect in a trial to lack of intervention effectiveness, when in reality it resulted from poor implementation [32].

Despite its importance, implementation fidelity in the context of interventions for epilepsy is almost never reported [33–35]. A range of psychosocial interventions have been developed and tested for epilepsy [33, 35], but only one assessment of treatment fidelity has, to our knowledge, been published [36].

The reasons for this are unknown. However, surveys of treatment outcome researchers in other fields [37–39] indicates potentially important barriers include a lack of knowledge about and awareness of treatment fidelity and a lack of credence currently given to such findings by journals.

Fidelity has been conceptualised and measured in various ways [31]. In terms of measurement, what is arguably most rigorous is for persons independent of the intervention to observe sessions and rate them.

One key element of implementation fidelity that needs to be rated is “adherence.” This is the extent to which the core content of a programme was delivered as instructed, including specific topics and techniques to use [40]. High adherence requires strictness to instructions and knowledge of how to deliver each component as required by the protocol.

Whilst adherence is often the only way treatment fidelity is assessed [41, 42], by itself it may not provide a comprehensive picture of intervention delivery as it does not account for “how” the content was provided. This is an oversight since a person may deliver an intervention's content as prescribed but do it with little competence. Low competence may affect intervention acceptance and subsequent performance of skills [43].

One aspect of competence which appears particularly important when delivering group-based complex interventions is the extent of interactivity between the facilitator and recipients, or in other words, the degree of “didacticism” [44–46]. Skinner et al. [44] determined the proportion of facilitator to participant talk during a group-based education intervention for diabetes and found that lower facilitator talk ratios predicted greater improvements in participants' beliefs about diabetes and in their metabolic control.

This may be the case because whilst some didacticism is required to ensure participants remain oriented to the goals of the intervention and certain information provided, interaction permits participants to share and learn from each other, empowers them, and means they ask questions and seek clarification to ensure the intervention is tailored to their needs.

For our intervention, it is not yet known what level of didacticism represents the optimum and is associated with the greatest improvement in patient outcomes. It is though important at this stage to gauge what balance between adherence and didacticism is being achieved.

### 1.1. Current Project

In this study we sought to

1. develop a measure of adherence for the intervention and evaluate its reproducibility
2. use an existing method for assessing didacticism and evaluate its reproducibility when applied to our intervention
3. then, using audio recordings of intervention sessions, describe the extent of adherence and didacticism demonstrated in the delivery of the intervention in the context of the pilot RCT

In presenting this study, we also sought to provide a rare practical example of how outcome researchers in neurology can readily develop, test, and use simple measures of treatment fidelity to provide informative assessments.

## 2. Material and Methods

### 2.1. Study Setting

The pilot RCT recruited PWE from 3 hospital EDs in North-West England. Patient inclusion criteria were as follows: being ≥16 years of age, having a documented diagnosis of epilepsy, having visited ED on ≥2 occasions in the previous 12 months, and being prescribed antiepileptic medication. Patients with all epilepsy syndromes and all types of focal and generalised seizures were permitted to participate.

The trial ultimately enrolled 53 participants; 26 were randomized to the intervention. Ages ranged from 18 to 69 years; median time since diagnosis was 16.8 years. We are not able to describe the participants' actual type of epilepsy or seizures. This was because recruitment occurred within EDs, rather than from neurology departments. Little information was recorded within participants' ED records about their epilepsy and when information was recorded, it was done so according to differing classification systems.

The National Research Ethics Committee North West—Liverpool East approved the study (15/NW/0225). Informed consent was obtained from all participants.

### 2.2. The Intervention

The intervention was developed to be delivered to groups of up to 10 patient-carer dyads by a single facilitator with knowledge of epilepsy, like a specialist epilepsy nurse [30]. It contains 6 modules. See Table 1 for further details.

To help standardise the intervention, delivery follows a detailed trainer's manual. This provides the content to be covered and outlines the teaching techniques to be used at different stages.

Materials include presentation slides, videos illustrating seizure types, the recovery position, and first aid. Patients get to take copies of the slides and additional information booklets away with them and can access a website with the intervention content on.

### 2.3. Training of Facilitator and Intervention Delivery in Trial

For the purposes of the pilot RCT, a single facilitator, recommended by the UK's National Society for Epilepsy, delivered the intervention within the education centre of a local teaching hospital. The facilitator was a registered nurse with 30 years of experience (18 months as an epilepsy nurse). An administrator was also present at each course to take a register and organise room layout and refreshments.

The facilitator's training consisted of them familiarising themselves with the facilitator manual, delivering 2 practice courses with PWE, and carers not participating in the trial and receiving feedback on this from the intervention development team.

All courses were audio-recorded using digital-orbital microphones. The facilitator was aware that these recordings were to be listened to and rated for fidelity.

### 2.4. Developing the Intervention Fidelity Measurement Instruments

#### 2.4.1. Adherence

To measure adherence, a checklist of the intervention's intended content was developed on the basis of the facilitator's manual (Table 1). It listed the 37 items to be delivered across the intervention's 6 modules. The checklist asked a rater to report, using a 0-2 ordinal scale, the extent to which each item was delivered (0 = item not delivered, 1 = partially delivered, and 2 = fully delivered).

The number of items within the modules differs (range of items within modules = 4-10). To allow adherence within the different course modules to be compared, average adherence ratings were calculated.

#### 2.4.2. Competence

Following the method developed by Wojewodka et al. [36], didacticism was assessed using the Eudico Linguistic Annotator (ELAN) 5.1 software [47]. It permitted a rater to listen to the audio recording of a course and simultaneously code when the facilitator was speaking. The total amount of facilitator speech, as a proxy measure of how “didactic” the course, was then calculated. This was divided by the duration of the course to generate the percentage of course time during which the facilitator was speaking. Filler words (e.g., “oh,” “okay,” and “yeah”) were not considered instances of facilitator speech.

#### 2.4.3. Testing the Measures

To assess reliability of the fidelity measures, two raters independent from the trial and intervention teams individually evaluated each course using the fidelity measures. Raters were final year students completing a British Psychological Society accredited, Bachelor of Science psychology degree. Their rating training consisted of them familiarising themselves with the intervention materials and completing practice adherence and didacticism ratings on two courses not delivered as part of the trial.

### 2.5. Data Analysis

#### 2.5.1. Testing the Measures

To provide a measure of response burden for the different fidelity measures, the average duration of the courses was calculated along with the average time it took a rater to asses them using the different measures.

The intraclass correlation coefficient (two-way random effects, absolute agreement, and multiple raters) [48] was used to test the agreement between the two raters' didacticism ratings, with the following cutoffs being used: <0.40 = poor agreement, 0.40–0.59 = fair, 0.60–0.74 = good, and > 0.74 = excellent agreement [49].

For the adherence measure, the ratings from the two raters were tabulated and simple percentage agreement was first calculated. Interrater reliability was then assessed using the chance-corrected weighted kappa statistic. A kappa value of 0.81–1.00 was considered to indicate almost perfect agreement, 0.61–0.80 substantial agreement, 0.41–0.60 moderate agreement, 0.21–0.40 fair agreement, and 0.00–0.20 slight agreement. Since paradoxical values of kappa can, though, occur because of bias or skewed prevalence [50], the influence of these factors was considered by calculating a prevalence index (PI) and a bias index (BI) and by comparing the change in kappa when the prevalence-adjusted bias-adjusted kappa (PABAK-OS) was calculated. PI can range from −1 to +1 (0 indicates equal probability), whilst BI ranges from 0 to 1 (0 indicates equal marginal proportions and so no bias) [51].

#### 2.5.2. Course Fidelity

The raters' adherence and didacticism scores for each course were averaged and described using descriptive statistics.

Unadjusted linear regression (with robust standard errors) was completed to explore the association between the adherence rating for a course and the didacticism rating. The beta coefficient (*β*) and 95% confidence interval (CI) is reported.

Interrater agreement was calculated using MedCalc 18.2.1, regression was completed using STATA 11, and prevalence-adjusted bias-adjusted kappa was calculated using the PABAK-OS calculator (http://www.singlecaseresearch.org/calculators/pabak-os).

## 3. Results

### 3.1. Intervention Courses

Ultimately 20/26 of the PWE randomized to the intervention attended a course, with the facilitator delivering 7 courses over ~7 months. Course characteristics are in Table 2.

The average time for each course was 152 minutes (SD = 28.8) (excluding break periods). The average time it took one rater to complete an adherence rating for a course was 135 minutes (SD 36.6). The average time it took a rater to complete an assessment of didacticism was 308 minutes (SD = 49.62).

### 3.2. Evaluating the Fidelity Instrument

#### 3.2.1. Interrater Reliability: Adherence

For 96% of adherence items, the two raters made the exact same judgement with regards to the extent to which the item was delivered, but the weighted kappa statistic was only 0.66 (95% CI 0.50 to 0.83). This paradox is accounted for the large difference in probability of the different categories being used and a consequent prevalence bias (PI = –0.83; BI = 0.06). Specifically, the two raters used the category “fully delivered” to a much greater extent than they did the remaining categories “partially delivered” or “not delivered at all”; indeed, 94.6% of their ratings used the category “full delivered” (Supplementary Table 1). Given this, the PABAK-OS statistic of 0.91 (95% CI 0.85, 0.97) likely provides a more accurate estimate of actual concordance between raters.

#### 3.2.2. Interrater Reliability: Didacticism

With a coefficient of 0.96 (95% CI 0.78, 0.99), the intraclass correlation coefficient indicated that the two raters' judgement with regards to didacticism was highly correlated.

### 3.3. Evaluating Course Fidelity

#### 3.3.1. Adherence Results

Adherence was found to be high. Of the 259 items meant to be delivered across the 7 courses, 228 (88.0%) were fully delivered and only 8 (3.1%) were judged to have not been delivered at all. The average adherence rating given to the items in the courses was 1.88 (SD = 0.11, range 1.65 to 1.97) (Table 2).

When looking at the adherence ratings given to the different course modules, module 5 had the highest proportion of its items across the 7 courses fully delivered (i.e., 100% of them). Module 3 had the lowest amount (i.e., 71.4%) (Supplementary Table 2).

The mean and range of adherence scores given to the individual intervention items shows no item proved too challenging to be fully delivered at least once. The mean score of only one intervention item—namely that requiring the facilitator to inform the participants about when the demonstrated recovery position should and should not be delivered—fell below 1 (i.e., 0.79).

#### 3.3.2. Didacticism

The mean percentage of facilitator speech across the courses was 55% (SD = 5.4), with a range of 49 to 64%.

Regression analysis indicated that adherence and didacticism were associated (*β* = 26.6, 95% CI 3.35, 49.88), with increasing adherence being associated with greater facilitator speech within course. Adherence and didacticism shared 28% of variance (*R* ^2^ = 0.2838).

## 4. Discussion

### 4.1. Main Findings

This study is aimed at developing a measure of adherence for our intervention and using an existing measure of didacticism to assess the level of intervention fidelity achieved during the delivery of a seizure first aid intervention in a pilot RCT setting. Overall, the results suggest that the intervention was feasible and delivered as intended across the trial by the facilitator.

The checklist adherence measure indicated that the facilitator delivered almost all the items prescribed by the treatment manual. Across the courses, only 8 of the intended 259 items were not fully delivered. Moreover, no single item was found not to have been fully delivered at least once.

Increasing adherence can, though, potentially comprise competence [52]. The intervention was developed with the assumption that interactivity is key. Our results indicate that the facilitator was able to achieve a high adherence to the treatment protocol, as well as permit extensive interaction. Across the courses, they spoke, on average, 55% of the time.

These findings indicate that the estimates of the intervention's effects that will be produced by the pilot RCT in due course can be interpreted as accurate impressions of its benefits or otherwise.

### 4.2. Implications

Our results have implications for fidelity measurement within a future definitive trial.

Firstly, raters can, with only modest training, give reliable fidelity assessments. Raters agreed ~95% of the time on the adherence scale and showed high agreement on the didacticism measure. This indicates the checklist promotes a common understanding between raters about the criteria they are judging the courses against. In terms of the didacticism measure, the high agreement is likely attributable to the use of the computer software and that audio recordings were of sufficient quality to allow the raters to distinguish facilitator from non-facilitator speech.

In the context of full RCTs, Wojewodka et al. [36] and Mars et al. [53] used adherence instruments comparable to ours to evaluate broader self-management interventions for epilepsy and pain, respectively. Their raters agreed 80% of the time. The higher absolute agreement in our study may be attributable to the level of detail provided by our checklist and that we only had 7 courses to rate and so fewer items were assessed for agreement (e.g., 285 items vs. 425 in [36]). For didacticism, we used the same ELAN-based approach developed by Wojewodka et al. [36]. Our raters demonstrated the same level of agreement as theirs (i.e., ICC 0.96 in our study vs. 0.97 in theirs).

A second important implication of our study findings relates to the resources required to complete a fidelity assessment. Medical Research Council publications [29] note the importance of evaluating fidelity. Minimal guidance is, however, provided regarding how to do it. Intervention developers say this is one reason why they fail to assess fidelity [37]. We here provide a rare practical example on how to develop a simple measure of adherence, use an existing measure of didacticism, and establish reliability to provide an informative assessment. This could be used by teams planning similar evaluations and create an awareness amongst funders of what is required. On average, one of our intervention sessions lasted 152 minutes. It took though, 443 minutes to assess a course for adherence and didacticism.

Should our intervention ultimately be used in clinical practice, services will need measures to allow them to regularly check the quality with which they are delivering the intervention. Given the time they require to complete, our measures may not be ideal. What opportunities therefore exist to make the process more time efficient? Do both the adherence measures, for instance, need to be used? The results from our exploratory regression analysis indicate that adherence and didacticism are only moderately associated and so appear to be capturing different elements of fidelity. Thus, for a comprehensive fidelity evaluation, both measures are needed.

The approach we used to rate didacticism was particularly time consuming. Alternative approaches include participants or the facilitator rating delivery. Whilst potentially quicker, such approaches are not ideal. The former can be liable to floor effects (with patients appearing to be unwilling to rate therapist delivery poorly) [54], whilst therapists can overestimate their performance compared to independent ratings [55]. Some reduction in time could though come from reducing the number of adherence items. We asked raters to rate all courses for the presence of all the items that together formed the intervention. Currently, it is not known which items comprise its active, behaviour-changing ingredients nor how they interact. Future experimental work and interviews with recipients could help determine what these are and allow a more abbreviated adherence checklist to be used.

### 4.3. Strengths and Weaknesses

Strengths include that all courses were assessed and that the assessments were completed by persons not involved in the trial. The latter helped maintain independence and minimise bias. There are, though, potential limitations. Firstly, as this was a pilot trial, only one person delivered the intervention. It remains to be determined how well our findings generalise to other facilitators.

Secondly, our findings do not tell us how well the treatment can be delivered when group sizes are larger. We planned for the intervention to be delivered to 8-10 persons. In the pilot, the average group size was though 5.

Thirdly, with only 7 courses delivered, our sample size was small. To express the uncertainty this brings to the precision of the estimates our study provides, 95% CI are reported.

Finally, audio recordings formed the basis of our fidelity assessment. This worked well in our study when assessing the delivery of core items from a checklist and was unobtrusive. However, it is possible that such recordings may not capture all the subtleties of facilitator competence involving nonverbal behaviours and the dynamics of facilitators as well as individual and group interactions. This needs to be taken in account when considering our measure of didacticism.

## 5. Conclusions

We can be confident that the intervention was delivered with high levels of adherence and competence within the pilot RCT, and so we anticipate that our estimate of intervention effect will not be influenced by poor implementation fidelity. In presenting a rare worked example of how adherence and competence can be assessed, we anticipate that this study could help promote increased assessment and reporting of treatment fidelity when assessing complex interventions for epilepsy.

## Figures and Tables

**Table 1:** Adherence items for intervention.

Modules	Description	Items	Points to mention for full delivery
(I) Orientation & behaviour change optimisation	Rules regarding confidentiality, quiz about common epilepsy myths, expectations and self-affirmation exercise completed	(1) Welcome	Facilitator welcomes group
		(2) Goals of this course	Facilitator outlines them
		(3) What would you like from today?	Facilitator provides opportunity for participants to share their expectations of course
		(4) True or false?	Presents 3 quiz questions to the group (any order is okay)
		(5) Taking on information (kindness questionnaire)	Participants asked to do a questionnaire

(II) Basic epilepsy & first aid knowledge	Professional video narrated by neurologist showing seizure types and applicable first aid; subgroups work to find answers to different questions concerning seizure first aid from selection of cards and present these. Designed to elicit participant beliefs and fears and for these to be discussed. Simple guidance given about when to call ambulance and management of postictal states and injuries	(1) Epilepsy, seizures, & how the brain works	Facilitator plays video
		(2) First aid for convulsive seizures exercise	Participants are asked to do the exercise relating to this topic
		(3) What can you do to help someone during a seizure?	Mention, in any order, all the following: (i) Look around—make sure it is safe (ii) Stay calm & stay with them (iii) Allow seizure to happen (iv) Check the time—if shaking does not stop after 5 minutes, dial 999 (v) Protect head (vi) Loosen any tight clothing around the neck (vii) Look for an epilepsy ID (viii) Stop people crowding
		(4) What not to do during a seizure	Mention, in any order, all the following. Do not (i) Hold them down (ii) Put something in the mouth (iii) Move them (unless dangerous) (iv) Give something to eat or drink (v) Try to bring them around
		(5) What to do after the seizure has stopped	Mention, in any order, all the following: (i) Check breathing (ii) Put in recovery position (iii) Minimise embarrassment (iv) Stay calm & stay with them (v) Look for injuries (vi) Make note of what happened (vii) Person will not usually need to go to the hospital
		(6) Questions or comments?	Facilitator provides opportunity for questions
		(7) Postseizure states	Mention, in any order, all the following: (i) Postictal state is a medical term for the recovery period immediately after a seizure (ii) Gives examples of some symptoms during this period (e.g., changes in awareness, senses, emotional, thoughts, and physical) (iii) Highlights if in doubt about what to do or if normal, seek medical assistance
		(8) Injuries	Mention, in any order, all the following: (i) Acknowledges possibility of injuries (ii) Directs participants to other resources for guidance on management
		(9) When to call an ambulance?	Acknowledges appropriateness to seek medical attention in all the following circumstances (any order is okay): (i) When shaking/seizure lasts more than 5 mins (may be referred to as “status epilepticus”) (ii) When one seizure follows another with no recovery (may be referred to as “cluster seizures”) (iii) If someone has difficulty breathing once shaking stopped (iv) If badly injured themselves (v) If seizure happened in water (vi) If it is their first ever seizure (vii) If you believe they need medical help
		(10) Questions or comments?	Facilitator provides opportunity for questions

(III) Recovery position	Professionally produced video and step-by-step slides of recovery position. Participants then work in pairs to practice recovery position with feedback from the facilitator	(1) Recovery position	Facilitator notes if a person is unconscious or asleep but breathing after a seizure, and it is not thought that the person has damaged neck or back, then they should be placed in recovery position
		(2) Recovery position	Facilitator plays video
		(3) Let us practice the recovery position	Recovery position practiced by at least one participant (the participant might take on role of playing the patient or the person putting the person in the recovery position)
		(4) Questions or comments?	Facilitator provides opportunity for questions

(IV) Informing others about epilepsy & how to help if seizures occur	Facilitated discussion about the different groups of people who might be of assistance when a seizure occurs, what information they need to know, how to help, and how to get this information to them (for example, you need to know how to help and how to get this information to these different groups)	(1) Who needs to know how to help?	Group asked to think about people around the patient that might need to be able to help if a seizure occurs; some examples given (e.g., family, friends, colleagues, public, and health professionals) by facilitator or participants
		(2) What they need to know & why	Mention, in any order, all the following: (i) That you have epilepsy (ii) What sort of seizures are normal for you (iii) What to do & not to do (iv) How you want to be helped (e.g., any preferences you have)
		(3) How to get this information to them. Family, friends & work colleagues	Facilitator invites suggestions from group or presents some possibilities (e.g., sharing information from course, encourage them to visit online resources, and download “how to help app”)
		(4) How to get this information to them. Members of the public and health workers	Facilitator invites suggestions from group or presents some possibilities (e.g., carrying epilepsy ID, such as an “I have epilepsy” card, putting information on mobile phone emergency information sections, and medical jewellery)
		(5) Questions or comments?	Facilitator provides opportunity for questions

(V) Medical ID, seizure triggers & home safety	Participants presented with 2 illustrated patient case stories. They are asked to consider what the patient in the story might have done to have achieved a more favourable outcome	(1) Personal stories—introduction	Facilitator introduces section (e.g., “we are now going to look at some personal stories…”)
		(2) Ben's story	Facilitator reads case story to group
		(3) How to change what happened to Ben?	Facilitator asks group for suggestions about how to change the outcome in Ben's story. Facilitator or participants mention, in any order, all the following: (i) The carrying of medical identification (ID) (ii) Paying attention to one's triggers for seizures (iii) Declining transportation to the hospital
		(4) Triggers	Gives examples of triggers
		(5) Knowing your triggers	Discuss importance of knowing one's triggers
		(6) Some ways of dealing with triggers	Gives some suggestions about how to identify and manage triggers
		(7) Sandra's story	Facilitator reads case story to group
		(8) How to change what happened to Sandra (warning signs; home safety)	Facilitator asks group for suggestions about how to change the outcome in Sandra's story. All the following should be mentioned (in any order): (i) Warning signs (aura) (ii) Home safety

(VI) Summary and consolidating learning	Key take-away messages from intervention for different participant categories outlined; directed to additional sources of information and provided with online access to course materials	(1) Main points to remember, if you have epilepsy:	Mention, in any order, all the following: (i) Epilepsy is common (ii) Whilst frightening, most seizures are short and stop by themselves (iii) Will not usually need emergency medical attention (iv) You can tell those around you how they can help (v) Tell friends and family how to deal safely with seizures (vi) Carry medical ID (vii) You may be able to reduce seizures and injury (viii) Think about things you could do differently
		(2) Main points to remember, if you know someone with epilepsy:	Mention, in any order, all the following: (i) Seizures can be upsetting but try to stay calm (ii) You have power to help (iii) Person is usually not in pain and will not remember (iv) Most seizures are short and will stop by themselves (v) Do not restrain the person or put anything in the mouth (vi) Usually the person will not need medical help, just reassuring and putting in the recovery position (vii) Time the seizure, if shaking lasts longer than 5 minutes, one seizure follows another, or the person has badly injured themselves call for an ambulance
		(3) Sources of further information	Facilitator notes information available from elsewhere (facilitator gives examples such as Epilepsy Society, Epilepsy Action, and NHS Choices)
		(4) What is on the back table and accessing the study website	Facilitator notes: (i) Additional information for participants to take away on table (ii) Directs participants to website containing the course materials
		(5) Questions or comments?	Facilitator provides opportunity for participants to ask questions or make comments (this might be in the manner of how well did this course meet your expectations of what you wanted it from it)

**Table 2:** Characteristics of the courses.

Course number	Course duration (excluding breaks)	Participant number	Adherence rating		Didacticism rating—ELAN—percentage of time facilitator speaking
			No. of items fully delivered (%)	*M* rating across 37 items (SD)	
1	189.00	5.00	35 (94.6%)	1.97 (0.11)	54.45%
2	182.00	8.00	33 (89.2%)	1.91 (0.35)	57.57%
3	166.00	6.00	33 (89.2%)	1.92 (0.25)	63.94%
4	112.00	6.00	35 (94.6%)	1.95 (0.22)	54.48%
5	154.00	4.00	32 (86.5%)	1.86 (0.40)	49.59%
6	126.00	2.00	27 (73.0%)	1.65 (0.69)	48.87%
7	137.00	4.00	33 (89.2%)	1.88 (0.39)	59.42%
Across 7 courses			228 (88.0%)	1.88 (0.11)	55.47% (SD = 5.35)

*Notes*. Each course consisted of 6 modules. Together, these contained 37 items that were to be delivered. The extent of each of these items delivered was rated using the following scale: 0 = item not delivered, 1 = item partially delivered, and 2 = item fully delivered.

## Data Availability

In the absence of a registry for such data, anonymous fidelity data is available upon request from the corresponding author. Original raw audio recording data from this trial is though not publicly available, as it contains confidential information.

## Supplementary Materials

Supplementary Materials Supplementary Table 1: agreement in adherence rating level between first and second raters. Supplementary Table 2: adherence ratings for each checklist item and module.

## References

[1] Casado V. (2011). Neurological patient care in emergency departments. A review of the current situation in Spain. *Neurología*.

[2] Jacoby A., Buck D., Baker G., McNamee P., Graham-Jones S., Chadwick D. (1998). Uptake and costs of care for epilepsy: findings from a U.K. regional study. *Epilepsia*.

[3] Royl G., Ploner C. J., Möckel M., Leithner C. (2010). Neurologische Leitsymptome in einer Notaufnahme. *Nervenarzt*.

[4] House of Commons Committee of Public Accounts (2015). *Services to People with Neurological Conditions (HC 502)*.

[5] Ridsdale L., McCrone P., Morgan M., Goldstein L., Seed P., Noble A. (2013). Can an epilepsy nurse specialist-led self-management intervention reduce attendance at emergency departments and promote well-being for people with severe epilepsy? A non-randomised trial with a nested qualitative phase. *Health Services and Delivery Research*.

[6] Noble A. J., Goldstein L. H., Seed P., Glucksman E., Ridsdale L. (2012). Characteristics of people with epilepsy who attend emergency departments: prospective study of metropolitan hospital attendees. *Epilepsia*.

[7] Kitson A., Shorvon S., Group CSA (2000). *Services for Patients with Epilepsy*.

[8] Ryan J., Nash S., Lyndon J. (1998). Epilepsy in the accident and emergency department-developing a code of safe practice for adult patients. South East and South West Thames Accident and Emergency Specialty Sub-committees. *Journal of Accident & Emergency Medicine*.

[9] Reuber M., Hattingh L., Goulding P. J. (2000). Epileptological emergencies in accident and emergency: a survey at St James’s University Hospital, Leeds. *Seizure*.

[10] Dixon P. A., Kirkham J. J., Marson A. G., Pearson M. G. (2015). National Audit of Seizure management in Hospitals (NASH): results of the national audit of adult epilepsy in the UK. *BMJ Open*.

[11] Dickson J. M., Taylor L. H., Shewan J., Baldwin T., Grunewald R. A., Reuber M. (2016). Cross-sectional study of the prehospital management of adult patients with a suspected seizure (EPIC1). *BMJ Open*.

[12] National Society for Epilepsy (2012). *When to dial 999*.

[13] British Epilepsy Association (2013). *What to do when someone has a seizure*.

[14] Limm E. I., Fang X., Dendle C., Stuart R. L., Egerton Warburton D. (2013). Half of all peripheral intravenous lines in an Australian tertiary emergency department are unused: pain with no gain?. *Annals of Emergency Medicine*.

[15] NHS England (2017). *NHS outcomes framework indicators-Feb 2017 release*.

[16] National Clinical Guideline Centre (2011). *The epilepsies: the diagnosis and management of the epilepsies in adults and children in primary and secondary care*.

[17] Bardsley M., Blunt I., Davies S., Dixon J. (2013). Is secondary preventive care improving? Observational study of 10-year trends in emergency admissions for conditions amenable to ambulatory care. *BMJ Open*.

[18] Elliott J., Shneker B. (2008). Patient, caregiver, and health care practitioner knowledge of, beliefs about, and attitudes toward epilepsy. *Epilepsy & Behavior*.

[19] Institute of Medicine (US) Committee on the Public Health Dimensions of the Epilepsies (2012). *Epilepsy Across the Spectrum: Promoting Health and Understanding*.

[20] Ridsdale L., Kwan I., Cryer C. (1999). The effect of a special nurse on patients’ knowledge of epilepsy and their emotional state. Epilepsy evaluation care group. *British Journal of General Practice*.

[21] Ridsdale L., Kwan I., Cryer C., and the Epilepsy Care Evaluation Group (2000). Newly diagnosed epilepsy: can nurse specialists help? A randomized controlled trial. *Epilepsia*.

[22] Noble A. J. (2015). Unplanned hospital use by people with epilepsy: a lever by which to bring about increased self-management support?. *Epilepsy & Behavior*.

[23] Noble A. J., McCrone P., Seed P. T., Goldstein L. H., Ridsdale L. (2014). Clinical- and cost-effectiveness of a nurse led self-management intervention to reduce emergency visits by people with epilepsy. *PLoS One*.

[24] Noble A. J., Morgan M., Virdi C., Ridsdale L. (2013). A nurse-led self-management intervention for people who attend emergency departments with epilepsy: the patients’ view. *Journal of Neurology*.

[25] Ridsdale L., Virdi C., Noble A., Morgan M. (2012). Explanations given by people with epilepsy for using emergency medical services: a qualitative study. *Epilepsy & Behavior*.

[26] Bradley P. M., Lindsay B. (2008). Care delivery and self-management strategies for adults with epilepsy. *Cochrane Database of Systematic Reviews*.

[27] Lindsay B., Bradley P. M. (2010). Care delivery and self-management strategies for children with epilepsy. *The Cochrane Database of Systematic Reviews*.

[28] Noble A. J., Marson A. G., Tudur-Smith C. (2015). Seizure first aid training’ for people with epilepsy who attend emergency departments, and their family and friends: study protocol for intervention development and a pilot randomised controlled trial. *BMJ Open*.

[29] Moore G. F., Audrey S., Barker M. (2015). Process evaluation of complex interventions: Medical Research Council guidance. *British Medical Journal*.

[30] Snape D. A., Morgan M., Ridsdale L., Goodacre S., Marson A. G., Noble A. J. (2017). Developing and assessing the acceptability of an epilepsy first aid training intervention for patients who visit UK emergency departments: a multi-method study of patients and professionals. *Epilepsy & Behavior*.

[31] Bellg A. J., Borrelli B., Resnick B. (2004). Enhancing treatment fidelity in health behavior change studies: best practices and recommendations from the NIH Behavior Change Consortium. *Health Psychology*.

[32] Dobson D., Cook T. J. (1980). Avoiding type III error in program evaluation: results from a field experiment. *Evaluation and Program Planning*.

[33] Michaelis R., Tang V., Wagner J. L. (2017). Psychological treatments for people with epilepsy. *Cochrane Database of Systematic Reviews*.

[34] Modi A. C., Wagner J., Smith A. W., Kellermann T. S., Michaelis R. (2017). Implementation of psychological clinical trials in epilepsy: review and guide. *Epilepsy & Behavior*.

[35] Noble A. J., Reilly J., Temple J., Fisher P. L. (2018). Cognitive-behavioural therapy does not meaningfully reduce depression in most people with epilepsy: a systematic review of clinically reliable improvement. *Journal of Neurology, Neurosurgery and Psychiatry*.

[36] Wojewodka G., Hurley S., Taylor S. J. C., Noble A. J., Ridsdale L., Goldstein L. H. (2017). Implementation fidelity of a self-management course for epilepsy: method and assessment. *BMC Medical Research Methodology*.

[37] Perepletchikova F., Hilt L. M., Chereji E., Kazdin A. E. (2009). Barriers to implementing treatment integrity procedures: survey of treatment outcome researchers. *Journal of Consulting and Clinical Psychology*.

[38] Sanetti L. M. H., DiGennaro Reed F. D. (2012). Barriers to implementing treatment integrity procedures in school psychology research: survey of treatment outcome researchers. *Assessment for Effective Intervention*.

[39] McGee D., Lorencatto F., Matvienko-Sikar K., Toomey E. (2018). Surveying knowledge, practice and attitudes towards intervention fidelity within trials of complex healthcare interventions. *Trials*.

[40] Cross W. F., West J. C. (2011). Examining implementer fidelity: conceptualizing and measuring adherence and competence. *Journal of Children's Services*.

[41] Schinckus L., van den Broucke S., Housiaux M., Diabetes Literacy Consortium (2014). Assessment of implementation fidelity in diabetes self-management education programs: a systematic review. *Patient Education and Counseling*.

[42] Durlak J. A., DuPre E. P. (2008). Implementation matters: a review of research on the influence of implementation on program outcomes and the factors affecting implementation. *American Journal of Community Psychology*.

[43] Sharpless B. A., Barber J. P. (2009). A conceptual and empirical review of the meaning, measurement, development, and teaching of intervention competence in clinical psychology. *Clinical Psychology Review*.

[44] Skinner T. C., Carey M. E., Cradock S. (2008). Educator talk’ and patient change: some insights from the DESMOND (diabetes education and self management for ongoing and newly diagnosed) randomized controlled trial. *Diabetic Medicine*.

[45] Coffman J. M., Cabana M. D., Halpin H. A., Yelin E. H. (2008). Effects of asthma education on children's use of acute care services: a meta-analysis. *Pediatrics*.

[46] Stenov V., Henriksen J. E., Folker A. P., Skinner T. C., Willaing I. (2015). Educator talk ratio as a quality indicator in group-based patient education. *Health Education Journal*.

[47] Lausberg H., Sloetjes H. (2009). Coding gestural behavior with the NEUROGES-ELAN system. *Behavior Research Methods, Instruments, & Computers*.

[48] Koo T. K., Li M. Y. (2016). A guideline of selecting and reporting intraclass correlation coefficients for reliability research. *Journal of Chiropractic Medicine*.

[49] Fleiss J. L. (1981). *Statistical Methods for Rates and Proportions*.

[50] Feinstein A. R., Cicchetti D. V. (1990). High agreement but low kappa: I. The problems of two paradoxes. *Journal of Clinical Epidemiology*.

[51] Byrt T., Bishop J., Carlin J. B. (1993). Bias, prevalence and kappa. *Journal of Clinical Epidemiology*.

[52] Waltz J., Addis M. E., Koerner K., Jacobson N. S. (1993). Testing the integrity of a psychotherapy protocol: assessment of adherence and competence. *Journal of Consulting and Clinical Psychology*.

[53] Mars T., Ellard D., Carnes D., Homer K., Underwood M., Taylor S. J. C. (2013). Fidelity in complex behaviour change interventions: a standardised approach to evaluate intervention integrity. *BMJ Open*.

[54] Schoenwald S. K., Garland A. F., Chapman J. E., Frazier S. L., Sheidow A. J., Southam-Gerow M. A. (2011). Toward the effective and efficient measurement of implementation fidelity. *Administration and Policy in Mental Health and Mental Health Services Research*.

[55] Hogue A., Dauber S., Lichvar E., Bobek M., Henderson C. E. (2015). Validity of therapist self-report ratings of fidelity to evidence-based practices for adolescent behavior problems: correspondence between therapists and observers. *Administration and Policy in Mental Health*.

