# Supplementary material for: Assessing Treatment Fidelity within an Epilepsy Randomized Controlled Trial: Seizure First Aid Training for People with Epilepsy Who Visit Emergency Departments
Source: Behav Neurol. 2019 Feb 3;2019:5048794. doi: 10.1155/2019/5048794 (PMC6378079; doi:10.1155/2019/5048794)
Supplement: Supplementary Materials — Supplementary Table 1: agreement in adherence rating level between first and second raters. Supplementary Table 2: adherence ratings for each checklist item and module. [file 5048794.f1.docx]

**Supplementary Materials**

**Supplementary Table 1** Agreement in adherence rating level between first and second raters

| **Rater 2** | **Rater 1** | | | **Total** |
| --- | --- | --- | --- | --- |
|  | 0 = Item not delivered | 1 = Item partially delivered | 2 = Item fully delivered |  |
| 0 = Item not delivered | 8 | 0 | 0 | 8 |
| 1 = Item partially delivered | 0 | 6 | 1 | 7 |
| 2 = Item fully delivered | 3 | 13 | 228 | 244 |
| **Total** | 11 | 19 | 22 | 259 |

**Supplementary Table 2** Adherence ratings for each checklist item and module

| **Module** | **Mean adherence rating for module across courses** *(SD, range)* | **Item** | **Mean adherence rating for item across courses**  *(range)* |
| --- | --- | --- | --- |
| **Orientation & behaviour change optimisation** | 1.98  (*SD*=0.08,  1.50-2.00) | 1. Welcome | 1.92 (1.50-2.00) |
|  |  | 1. Goals of this course | 2.00 (2.00-2.00) |
|  |  | 1. What would you like from today? | 2.00 (2.00-2.00) |
|  |  | 1. True or false? | 2.00 (2.00-2.00) |
|  |  | 1. Taking on information (Kindness Questionnaire) | 2.00 (2.00-2.00) |
| **Basic epilepsy & first aid knowledge** | 1.86  (*SD*=0.37,  0.00-2.00 | 1. Epilepsy, seizures & how the brain works | 2.00 (2.00-2.00) |
|  |  | 1. First aid for convulsive seizures exercise | 2.00 (2.00-2.00) |
|  |  | 1. What can you do to help someone during a seizure? | 2.00 (2.00-2.00) |
|  |  | 1. What not to do during a seizure | 2.00 (2.00-2.00) |
|  |  | 1. What to do after the seizure has stopped | 2.00 (2.00-2.00) |
|  |  | 1. Questions or comments? | 2.00 (2.00-2.00) |
|  |  | 1. Post-seizure states | 1.64 (1.00-2.00) |
|  |  | 1. Injuries | 1.07 (0.00-2.00) |
|  |  | 1. When to call an ambulance? | 1.92 (1.50-2.00) |
|  |  | 1. Questions or comments? | 2.00 (2.00-2.00) |
| **Recovery position** | 1.55  (*SD*=0.78,  0.00-2.00) | 1. Recovery position I | 0.79 (0.00-2.00) |
|  |  | 1. Recovery position II | 2.00 (2.00-2.00) |
|  |  | 1. Let’s practice the recovery position | 1.71 (0.00-2.00) |
|  |  | 1. Questions or comments? | 1.71 (0.00-2.00) |
| **Informing others about epilepsy & how to help if seizures occur** | 1.95  (*SD*=0.14,  1.50-2.00) | 1. Who needs to know how to help? | 1.93 (1.50-2.00) |
|  |  | 1. What they need to know & why | 2.00 (2.00-2.00) |
|  |  | 1. How to get this information to them. Family, friends & work colleagues. | 2.00 (2.00-2.00) |
|  |  | 1. How to get this information to them. Members of the public and health workers. | 2.00 (2.00-2.00) |
|  |  | 1. Questions or comments? | 1.86 (1.50-2.00) |
| **Medical ID, seizure triggers & home safety** | 2.00  (*SD*=0.00,  2.00-2.00) | 1. Personal stories - introduction | 2.00 (2.00-2.00) |
|  |  | 1. Ben’s story | 2.00 (2.00-2.00) |
|  |  | 1. How to change what happened to Ben? | 2.00 (2.00-2.00) |
|  |  | 1. Triggers | 2.00 (2.00-2.00) |
|  |  | 1. Knowing your triggers | 2.00 (2.00-2.00) |
|  |  | 1. Some ways of dealing with triggers | 2.00 (2.00-2.00) |
|  |  | 1. Sandra’s story | 2.00 (2.00-2.00) |
|  |  | 1. How to change what happened to Sandra (Warning signs; home safety) | 2.00 (2.00-2.00) |
| **Summary & consolidating learning** | 1.77  (*SD*=0.50,  0.00-2.00) | 1. Main points to remember, if you have epilepsy: | 1.93 (1.50-2.00) |
|  |  | 1. Main points to remember, if you know someone with epilepsy: | 2.00 (2.00-2.00) |
|  |  | 1. Sources of further information | 1.57 (0.00-2.00) |
|  |  | 1. What’s on the back table and accessing the study website | 1.50 (0.00-2.00) |
|  |  | 1. Questions or comments? | 1.86 (1.00-2.00) |
